# Identification of sex- and inflammation-associated heterogeneity in the mouse omentum

**DOI:** 10.3389/fimmu.2025.1670112

**Published:** 2025-10-10

**Authors:** Yi Ding, Shu-Yu Xiao, Jun-Jie Wang, Zhi-Yi Ren, Xiao-Cao Miao, Xian-Ting Ding, Xin Xing, Dong-Xue Li

**Affiliations:** ^1^ School of Biomedical Engineering, Shanghai Jiao Tong University, Shanghai, China; ^2^ State Key Laboratory of Systems Medicine for Cancer, Shanghai Cancer Institute, Ren Ji Hospital, School of Medicine, Shanghai Jiao Tong University, Shanghai, China; ^3^ Department of Chemistry, University College London, London, United Kingdom; ^4^ Shanghai Jiao Tong University Affiliated Sixth People’s Hospital South Campus, Shanghai, China

**Keywords:** omentum, peritoneum, single-cell RNA sequencing, inflammation, macrophages, sexually dimorphisms

## Abstract

The omentum is a critical intraperitoneal organ essential for peritoneal homeostasis, yet detailed characterization of its cellular composition remains limited by the lack of validated markers. Here, we employed single-cell RNA sequencing to systematically define cellular heterogeneity in naive and activated mouse omentum from both sexes. Our analysis identified previously uncharacterized immune and stromal cell subsets, including three macrophage subtypes with activation-dependent gene expression patterns, implying specialized roles in inflammation and immune regulation. Comparative analysis revealed marked transcriptional differences between omental and peritoneal macrophages, underscoring tissue-specific microenvironments. Additionally, sexually dimorphic gene expression in omental stromal cells correlated with peritoneal macrophage polarization, indicating sex-specific regulatory mechanisms. Critically, macrophages from omentum of female mice with ovarian cancer metastases showed unique gene signatures associated with tumor migration and invasion. Collectively, we provide the first comprehensive atlas of omental cell populations stratified by sex and activation state, offering novel insights into peritoneal immunity and identifying potential therapeutic targets for inflammatory and metastatic diseases.

## 1 Introduction

The greater omentum is a specialized visceral adipose tissue originating from mesothelial cells, anatomically connecting the stomach, spleen, pancreas, and colon in both humans and mice (1, 2). Historically, the omentum was merely viewed as a supportive structure for abdominal organs with limited recognition of its biological roles; however, recent studies have revealed its diverse and significant functions, including immune modulation, inflammatory responses, tissue repair, and tumor metastasis, thus earning it the descriptive title of the “abdominal policeman” (2, 3).

Accumulating research has demonstrated three primary functional roles of the omentum. Firstly, it can actively encapsulate inflammatory lesions within the peritoneal cavity, such as ruptured ovarian cysts, intestinal ulcers, and injuries caused by trauma or surgical procedures. Through local inflammatory responses, it facilitates angiogenesis, accelerates wound healing, and prevents the spread of infection (4). Secondly, the omentum exhibits remarkable reparative capacities in various organs, including liver regeneration, recovery from chronic kidney injury, amelioration of neurodegenerative diseases, and repair of ischemic cardiac injury; however, the exact mechanisms underlying these reparative processes remain unclear (5–7). Thirdly, the omentum serves as a prominent site for tumor metastasis, notably in ovarian cancer, wherein recruited neutrophils and macrophages actively promote tumor growth and dissemination through immune and metabolic pathways (8–11).

The multifunctionality of the omentum primarily arises from its complex cellular composition. In addition to mesothelial cells and adipocytes, the omentum contains specialized perivascular structures known as milky spots, which harbor abundant immune cells such as macrophages, lymphocytes, mast cells, and stromal cells, structurally resembling secondary lymphoid tissues (12–15). Although previous studies have preliminarily demonstrated that immune cells within milky spots regulate local immune responses via paracrine or exosomal mechanisms (16, 17), our comprehensive understanding of the omentum’s cellular composition, heterogeneity, and specific functional roles under pathological conditions remains limited. Critically, technical constraints, such as limited marker specificity and insufficient resolution of conventional methods, have hampered the effective identification of rare cell populations and intermediate cellular states, significantly impeding a full understanding of omental cellular composition and function.

The peritoneum and omentum are anatomically and immunologically closely interconnected. Omental stromal cells secrete key immunoregulatory factors, such as retinoic acid and IL-33, supporting the activation, survival, and migration of immune cells (including macrophages, B cells, and T cells) into and out of the peritoneal cavity. Previous studies have confirmed immune cell trafficking between the omentum and peritoneum, mediated by integrins, demonstrating critical interactions and exchanges between immune cell populations of these tissues (18, 19). Thus, comparing immune cell populations between the omentum and peritoneum is essential for comprehensively elucidating their distinct immune functions and interactions within peritoneal immune regulation.

Single-cell RNA sequencing (scRNA-seq) technology provides unprecedented resolution and accuracy in identifying rare cell types and transitional cellular states, overcoming the limitations inherent in traditional bulk sequencing approaches, thereby offering an effective solution to these challenges (20–22). In this study, we utilized scRNA-seq to systematically construct a comprehensive transcriptional atlas of omental cells in mice across different sexes and inflammatory conditions, clearly elucidating how sex and inflammation modulate cellular heterogeneity. Moreover, we compared analogous cell populations among the whole omentum, omental stroma, and the peritoneal cavity. Our findings not only provide new theoretical insights into peritoneal immune regulation and inflammatory dynamics but also highlight potential clinical implications by laying a foundation for developing novel therapeutic strategies and targets for peritoneum-associated inflammatory diseases and intraperitoneal tumor metastasis.

## 2 Results

### 2.1 Single-cell RNA sequencing defines diverse cellular subsets in male and female mouse omentum

The cellular and molecular composition of the greater omentum remains poorly characterized. To generate a comprehensive cellular atlas and investigate potential sex-related differences, the greater omenta were isolated from adult male and female mice (**Supplementary Figures 1A, B**). Histological examination using hematoxylin and eosin (H&E) staining clearly illustrated the structural organization of the omentum, including immune cell clusters known as milky spots (**Supplementary Figure 1D**). No notable histological differences between sexes were observed. Single-cell suspensions were subsequently prepared by enzymatic digestion with collagenase I (**Supplementary Figure 1F**), and scRNA-seq was performed using the 10x Genomics Chromium platform. After rigorous quality control filtering, a total of 8,827 cells from male mice and 9,875 cells from female mice were retained for further analysis.

**Figure 1:**
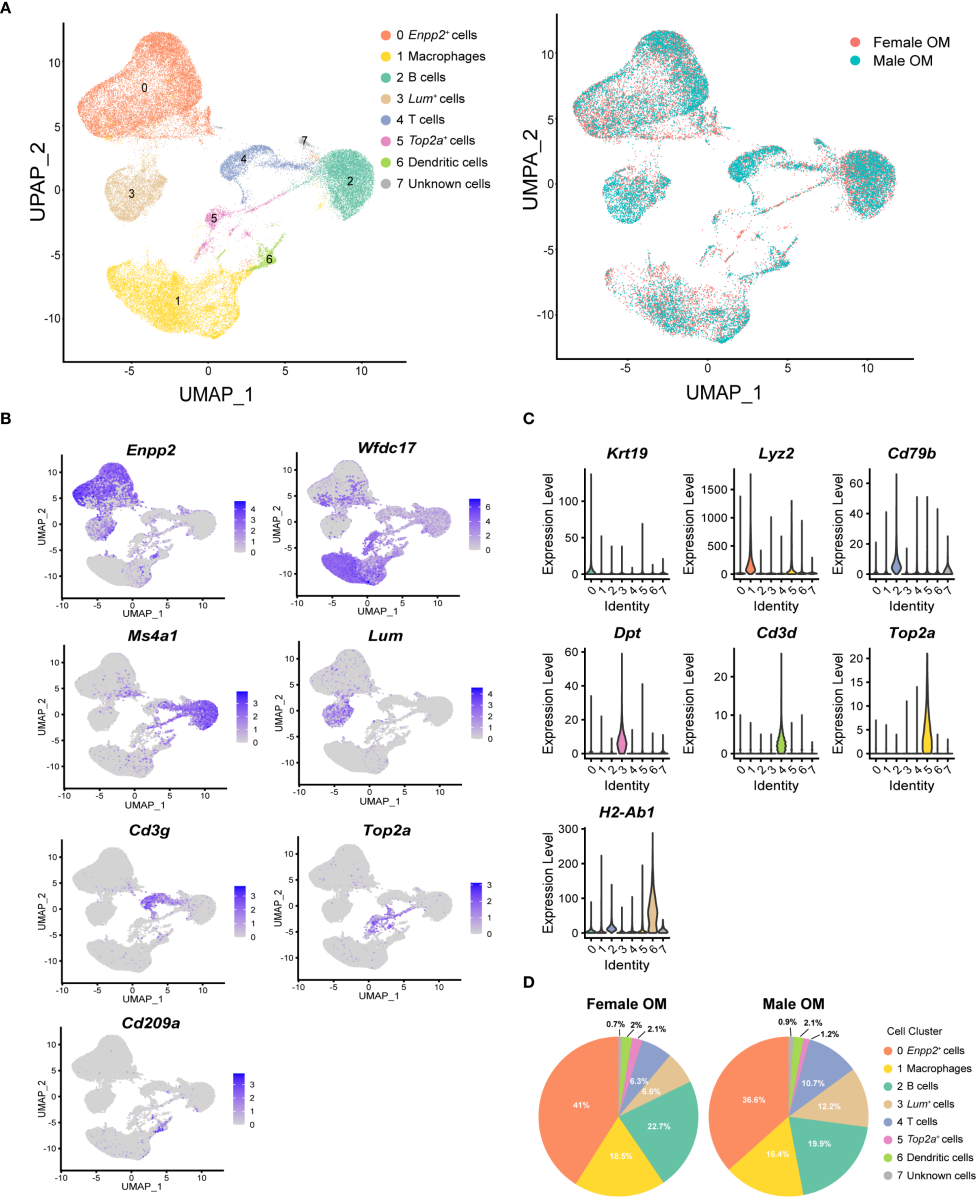
Single-cell RNA-sequencing reveals cellular heterogeneity of mouse omentum. **(A)** UMAP visualization of unsupervised clustering of single cells from mouse omentum. Each dot represents an individual cell, colored by cluster assignment according to annotated cell types (left) and sample origin (right). **(B)** UMAP plots showing the distribution of representative marker genes across eight identified clusters. Color scales indicate log-normalized gene expression levels. **(C)** Violin plots showing canonical marker gene expression across clusters. Highest log-normalized expression values are indicated for each cluster. **(D)** Proportions of the eight cell clusters in adult male (left) and female (right) mouse omentum.

Unsupervised clustering based on the Seurat algorithm identified several distinct cellular clusters, visualized using uniform manifold approximation and projection (UMAP). Eight major clusters were identified in both male and female omenta, comprising three non-immune and five immune cell subsets, distinguished primarily by the expression of the pan-immune marker gene protein tyrosine phosphatase receptor type C (*Ptprc*, also known as *Cd45*) (**Figure 1A**, **Supplementary Figure 2A**). Cell clusters were assigned biological identities based on known canonical marker genes and distinct gene expression signatures: ectonucleotide pyrophosphatase/phosphodiesterase 2 (*Enpp2*)^+^ stromal cells, WAP four-disulfide core domain 17 (*Wfdc17*)^+^ macrophages, membrane spanning 4-domains A1 (*Ms4a1*)^+^ B cells, lumican (*Lum*)^+^ stromal cells, Cd3 gamma subunit of T-cell receptor complex (*Cd3g*)^+^ T cells, DNA topoisomerase II alpha (*Top2a*)^+^ proliferative cells,CD209 antigen-like protein A (*Cd209a*)^+^ dendritic cells, and one cluster of unknown identity (**Figures 1B, C**, **Supplementary Figure 2B**). To validate these assignments and further delineate transcriptional profiles, we identified the top highly expressed marker genes within each cluster. For example, the key B-cell marker *Cd79b* was strongly expressed in the *Ms4a1* ^+^ B-cell cluster, while dermatopontin (*Dpt*) was prominently expressed in the *Lum* ^+^ stromal cell cluster, supporting the robustness of our classification approach (**Figure 1C**).

Subsequent comparative analysis of cell-type compositions revealed no significant differences between male and female mice. Consistent with previous reports highlighting the omentum’s role in facilitating immune cell trafficking into the peritoneal cavity (23, 24), macrophages and B cells were identified as the predominant immune cell populations within the omentum (**Figure 1D**, **Supplementary Figures 3A, B**). Consequently, subsequent analyses focused specifically on exploring gene expression heterogeneity within these macrophage and B-cell populations.

### 2.2 Gene expression profiling reveals distinct macrophage subsets within male and female mouse omentum

**Figure 2:**
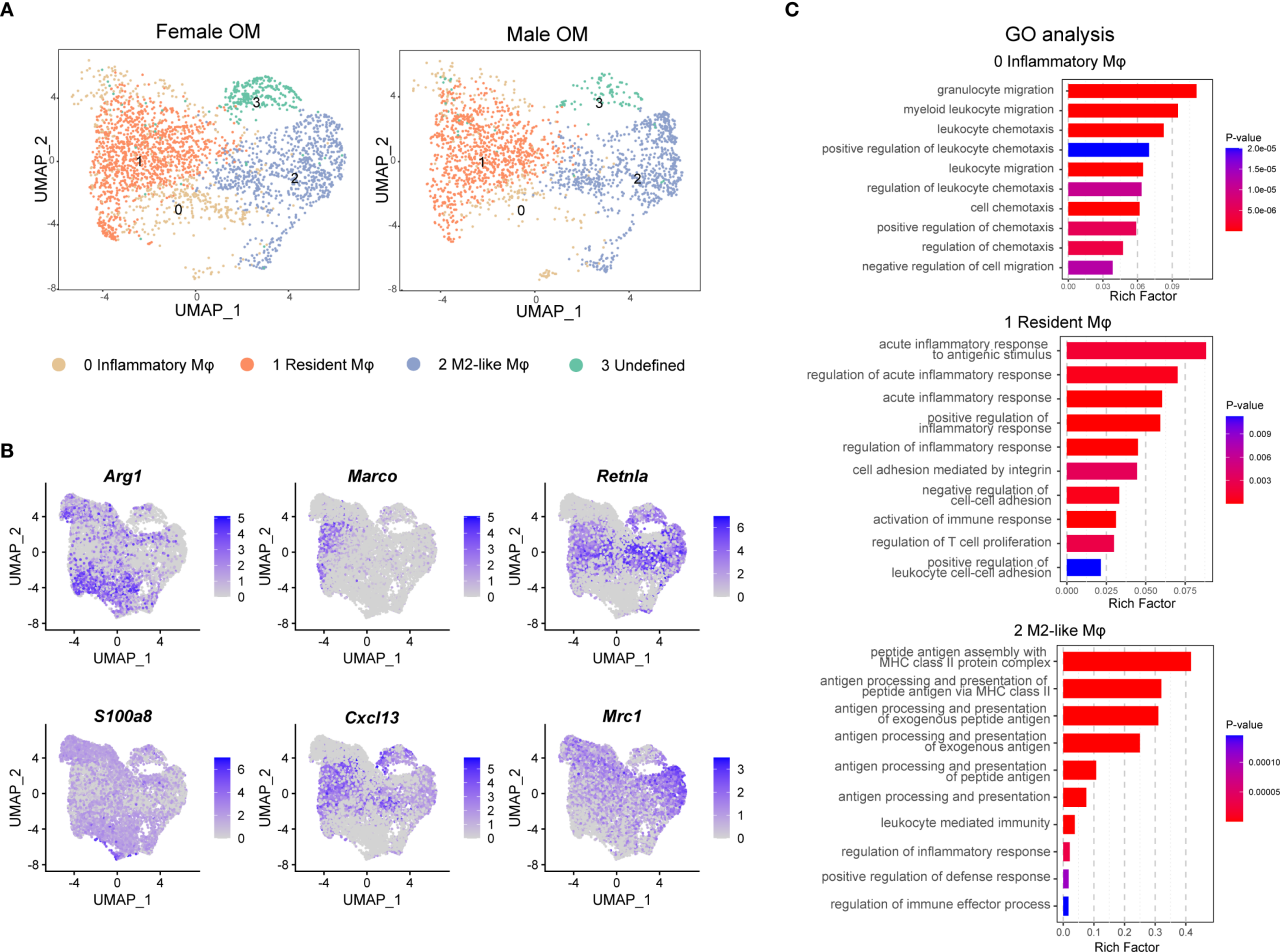
Identification of macrophage subtypes in male and female mouse omentum under naive conditions. **(A)** UMAP visualization of macrophage subpopulations in naive omentum from female and male mice. **(B)** UMAP plots highlighting expression patterns of marker genes defining four macrophage subtypes. Color scales indicate log-normalized gene expression levels. **(C)** Gene Ontology (GO) enrichment analysis showing functional differences among identified macrophage clusters.

Macrophages in nearly all tissues comprise distinct subpopulations with specialized functions, which are essential for regulating immune responses and maintaining tissue homeostasis. To further elucidate the molecular features of macrophages in the omentum, we conducted detailed subpopulation analyses using single-cell transcriptomics. Unsupervised UMAP clustering revealed three transcriptionally distinct macrophage subtypes within the omentum: inflammatory macrophages (cluster 0), resident macrophages (cluster 1), and M2-like macrophages (cluster 2), which were classified based on well-established macrophage marker genes (**Figure 2A**). Additionally, one smaller cluster (cluster 3) was identified as “undefined macrophages,” characterized by high expression of genes typically associated with stromal cells (e.g., *Enpp2*, decorin (*Dcn*), and collagen type i alpha 2 chain (*Col1a2*); **Supplementary Figure 4A**). Since the other three clusters shared clear and consistent macrophage marker expression (**Supplementary Figure 4B**), our subsequent analyses primarily focused on these three well-defined macrophage populations.

Further validation of marker genes demonstrated robust subtype-specific gene signatures: arginase 1 (*Arg1*) was highly expressed by inflammatory macrophages (cluster 0), macrophage receptor with collagenous structure (*Marco*) marked resident macrophages (cluster 1), and resistin like alpha (*Retnla*) characterized M2-like macrophages (cluster 2) (**Figure 2B**). Notably, resident macrophages also exhibited elevated expression of the B-cell chemokine c-x-c motif chemokine ligand 13 (*Cxcl13*), suggesting their potential roles in modulating omental milky spot formation and influencing local antibody-mediated immune responses (**Figure 2B**).

To gain deeper insight into the functional diversity of these macrophage subpopulations, we conducted Gene Ontology (GO) enrichment analysis on genes specifically enriched in each subtype. The results indicated distinct functional profiles: resident macrophages were primarily associated with processes involving cell adhesion and inflammatory responses, whereas M2-like macrophages showed enrichment in antigen processing and presentation pathways. In contrast, inflammatory macrophages exhibited pronounced involvement in cell migration pathways, underscoring their potential roles in chemotaxis and extravasation through blood vessels (**Figure 2C**).

### 2.3 Inflammatory conditions drive functional shifts among omental macrophage subpopulations in both sexes

**Figure 3:**
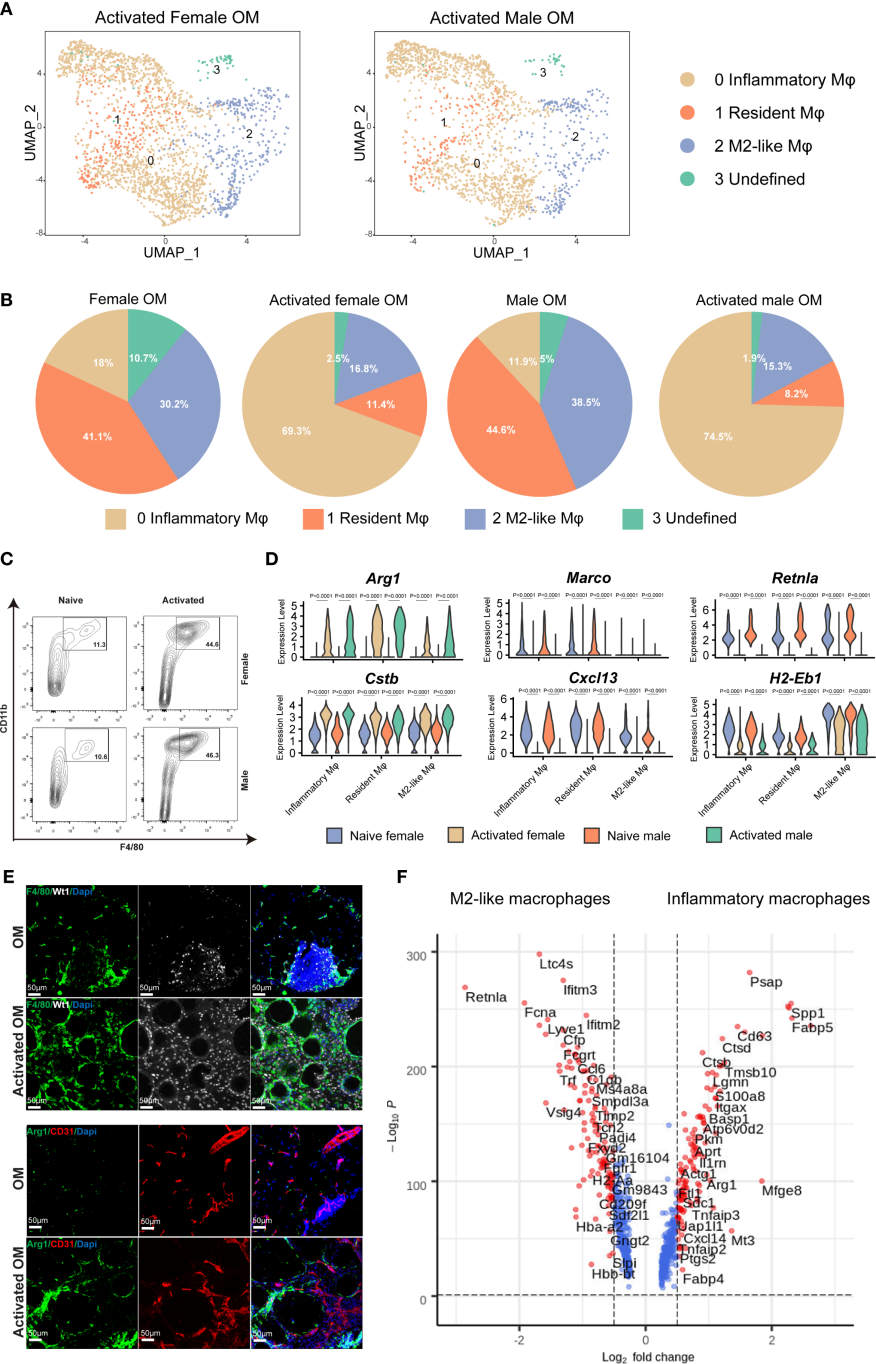
Dynamic changes in macrophage subtypes within mouse omentum following inflammatory activation. **(A)** UMAP plots of macrophages from activated omentum of female and male mice. **(B)** Quantitative comparisons of macrophage subtype proportions between naive and activated states in female and male mice. **(C)** Flow cytometric quantification of F4/80^+^ CD11b^+^ macrophages in CD45^+^ living cells from naive versus activated omentum across both sexes. **(D)** Violin plots showing expression levels of selected macrophage-specific marker genes in naive and activated omentum for both sexes. **(E)** IF staining illustrating the localization of total macrophages (F4/80^+^) and inflammatory macrophages (Arg1^+^) within mouse omentum under naive and inflammatory conditions. DAPI staining marks nuclei. Scale bar = 50 μm. **(F)** Volcano plots depicting differentially expressed genes (DEGs) between M2-like macrophages and inflammatory macrophages.

To elucidate the functional roles of omental macrophages during inflammation, we induced chronic peritonitis in mice by intraperitoneal injection of polydextran beads. After seven days, activated omenta were collected for histological and scRNA-seq analyses (**Supplementary Figure 1C**). H&E staining revealed no significant histological differences between male and female mice following inflammation, consistent with observations under physiological conditions (**Supplementary Figure 1E**). ScRNA-seq revealed similar macrophage subpopulations in both inflammatory omentum and physiological omentum states; however, significant differences were observed in the relative proportions of these subsets. Specifically, under inflammatory conditions, the frequency of inflammatory macrophages (cluster 0) markedly increased, whereas the proportions of resident macrophages (cluster 1) and M2-like macrophages (cluster 2) notably decreased (**Figures 3A, B**). Flow cytometry analysis further validated these findings, showing a significant increase in the percentage of CD11b^+^ F4/80^+^ macrophages following activation compared to naive controls, irrespective of sex (**Figure 3C**).

Upon inflammatory activation, we observed significantly increased expression levels of *Arg1* and cathepsin B (*Ctsb*), accompanied by significantly decreased expression of *Marco*, *Retnla*, and histocompatibility 2, class ii antigen e beta (*H2-Eb1*) compared to the naive condition (**Figure 3D**). This transcriptional shift indicates that macrophages undergo a clear functional transition from steady-state antigen presentation towards pro-inflammatory responses and tissue repair upon activation. Notably, the expression of the B-cell chemokine *Cxcl13* was significantly reduced specifically in the resident macrophage subset during inflammation, consistent with a reduced number of B cells in the inflamed omentum (**Figure 3D**, **Supplementary Figure 3A**). Furthermore, the notable decrease in *H2-Eb1* expression across macrophage subsets suggests a diminished antigen-presenting capacity in inflammatory conditions, further reinforcing the functional shift toward inflammatory effector roles.

Immunofluorescence (IF) staining of omental tissues demonstrated leukocyte infiltration marked by CD45 (**Supplementary Figure 5**). Wt1 staining specifically labeled mesothelial cells delineating the omental boundary, while CD31 staining clearly marked endothelial cells, outlining the internal vascular structures within the omentum. Additionally, F4/80 staining revealed a marked increase in macrophage numbers under inflammatory conditions, and Arg1-positive inflammatory macrophages were notably increased compared to the naive state (**Figure 3E**).

Lastly, comparative gene expression analysis between inflammatory and M2-like macrophages identified a specific set of inflammation-associated genes, including *s*100 calcium binding protein a8 (*S100a8*), secreted phosphoprotein 1 (*Spp1*), *Ctsb*, and integrin alpha 6 (*Itga6*), as significantly upregulated within inflammatory macrophages, further highlighting their critical roles in inflammation-mediated responses within the omentum (**Figure 3F**).

### 2.4 Tissue-specific transcriptional differences between omental and peritoneal macrophages

Given the close anatomical proximity and known immunological interactions between the omentum and peritoneal cavity (18, 19), we next compared cellular subpopulations between these two tissues to better understand tissue-specific differences and functional relationships. We compared the cell clusters identified in the total omentum, omental stroma, and PC. Cells isolated from the peritoneal cavity lacked stromal cell populations but exhibited a greater abundance of macrophages, B lymphocytes, and a substantial proportion of T lymphocytes (**Supplementary Figures 3A, B**). Furthermore, comparative analyses of cell clusters from total omentum and isolated omental stroma demonstrated highly similar clustering profiles, suggesting comparable cellular compositions and transcriptional signatures, albeit with varying proportions of specific cell types (**Supplementary Figures 3A, B**).

**Figure 4:**
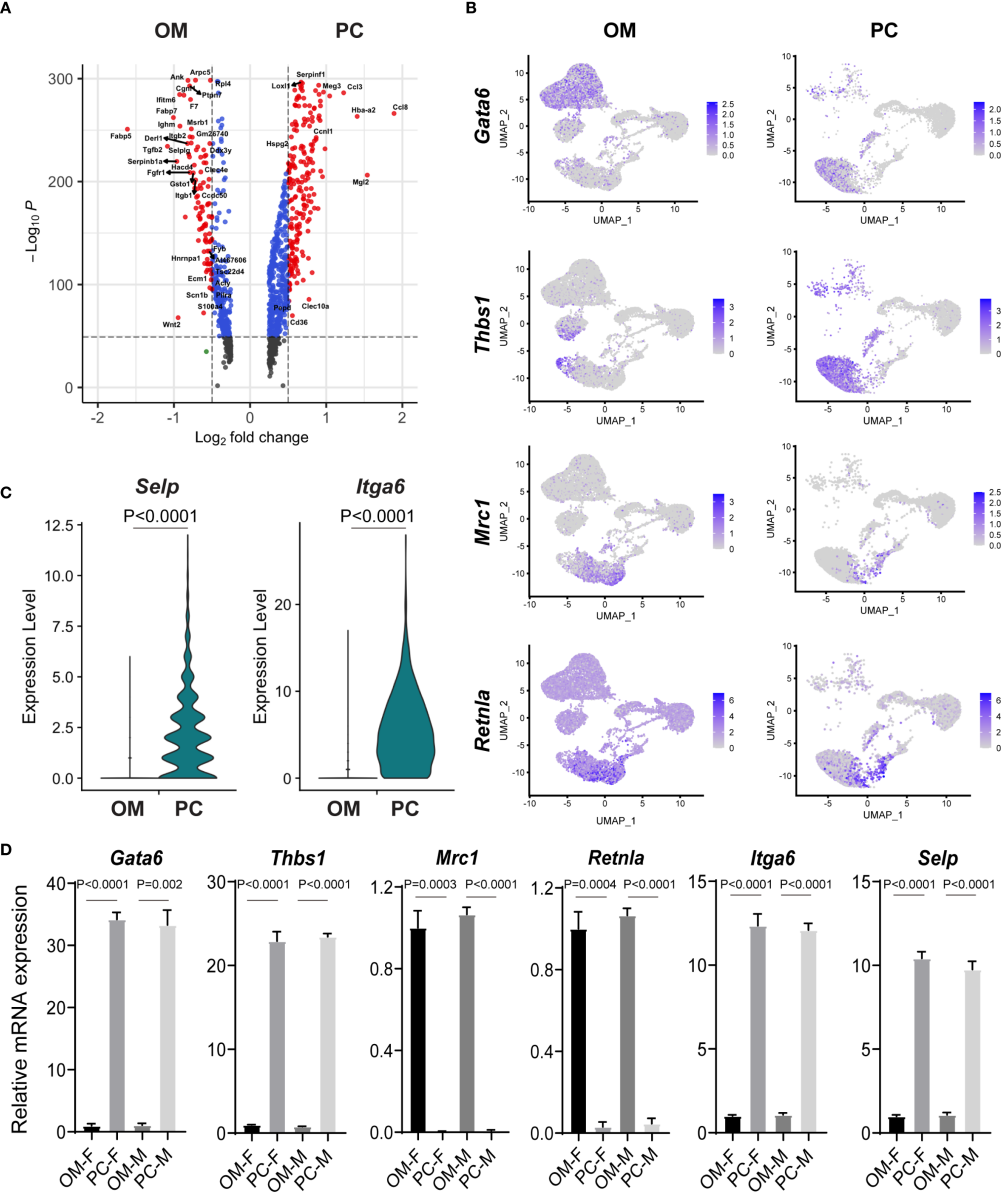
Comparative gene expression analysis reveals macrophage heterogeneity between omentum and peritoneal cavity. **(A)** Volcano plot illustrating differentially expressed genes (DEGs) between macrophages isolated from the omentum (OM) and peritoneal cavity (PC). Significant DEGs are highlighted based on log fold-change and statistical significance. **(B)** UMAP plots depicting expression patterns of selected macrophage-associated marker genes (*Gata6, Thbs1, Mrc1, Retnla)* between macrophages from OM and PC samples. **(C)** Violin plots comparing the mRNA expression levels of *Selp* and *Itga6* between macrophages isolated from OM and PC. **(D)** Quantitative RT-PCR analysis confirming expression patterns of macrophage-related genes (*Gata6*, *Thbs1, Mrc1, Retnla*, *Itga6*, and *Selp*) in male and female macrophages isolated from OM and PC tissues.

Although peritoneal macrophages are among the most extensively characterized resident macrophage populations, their functional and transcriptional relationship to omental macrophages remains unclear. To clarify this issue, we performed differential expression analysis between macrophages isolated from the omentum and peritoneal cavity (**Figure 4A**). Consistent with previous reports, our single-cell sequencing results confirmed the specific expression of gata binding protein 6 (*Gata6*) and thrombospondin 1 (*Thbs1*) in peritoneal macrophages but not in macrophages from the omentum. Interestingly, within omental tissue, *Gata6* expression was predominantly detected in *Enpp2* ^+^ stromal cells rather than macrophages (**Figure 4B**, **Supplementary Figures 6A**). Conversely, omental macrophages uniquely expressed classical M2-associated markers, including *Mrc1* (*Cd206*) and *Retnla*, aligning with previously reported characteristics of adipose tissue macrophages. Additionally, peritoneal macrophages exhibited elevated expression of *Itga6* and P-selectin (*Selp*), further reflecting their unique adaptation to the peritoneal microenvironment (**Figure 4C**).

To validate these findings, macrophages were isolated from omentum and peritoneal cavity, and gene expression levels of selected marker genes (*Gata6*, *Thbs1*, mannose receptor c-type 1(*Mrc1*), *Retnla*, *Itga6*, and *Selp*) were quantified by qRT-PCR. Consistent with our scRNA-seq data, *Gata6* and *Thbs1* showed significantly higher expression in peritoneal macrophages, whereas *Mrc1* and *Retnla* were markedly enriched in omental macrophages. Moreover, peritoneal macrophages expressed significantly higher mRNA levels of *Itga6* and *Selp* compared to their omental counterparts (**Figure 4D**). Notably, these expression patterns were consistent across both male and female mice, suggesting that the observed macrophage heterogeneity is primarily tissue-dependent rather than sex-dependent (**Figure 4D**).

### 2.5 Omental stromal cells contribute to sexual dimorphism in peritoneal macrophages

Recent studies demonstrated that female CD102^+^ peritoneal macrophages, regulated by ovarian-derived factors, express higher levels of genes related to lipid transport and immune defense. Interestingly, no significant sex-related differences were observed in omentum-derived CD102^+^ macrophages within bone marrow chimeric experiments (25). To further explore potential sex-related differences in omental macrophages, we analyzed our scRNA-seq data from male and female mice. Consistent with previous findings, no significant differences were detected in macrophage clustering patterns between sexes, suggesting that non-immune cells in the omentum might indirectly influence the observed sexual dimorphisms in peritoneal macrophages (**Figure 2A**).

**Figure 5:**
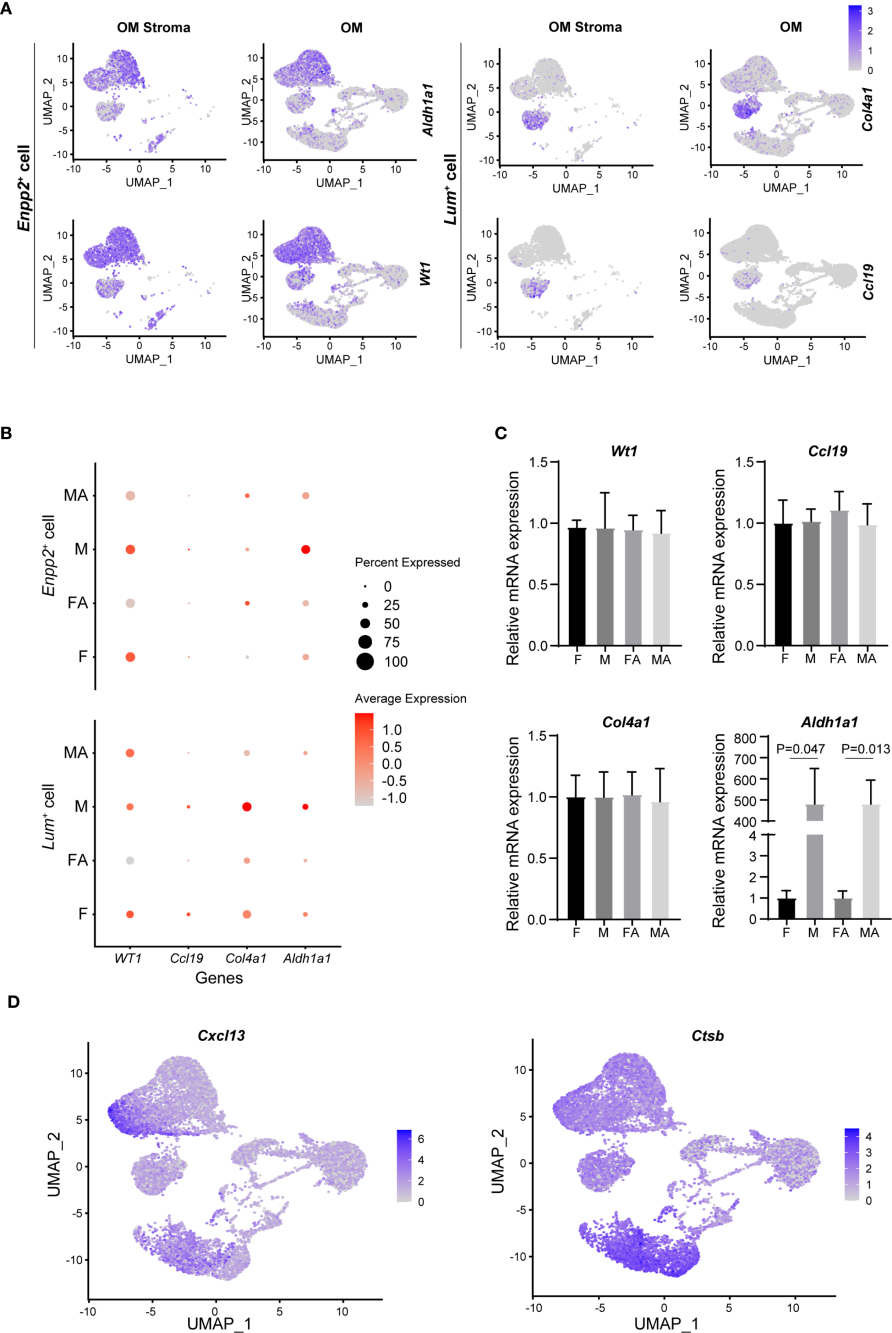
Stromal cells in mouse omentum contribute to sexual dimorphism of peritoneal macrophages. **(A)** UMAP visualization of *Aldh1a1*, *Wt1*, *Col4a1*, and *Ccl19* gene expression within omental stroma (OM stroma) and whole omentum (OM). **(B)** Comparison of mRNA expression levels of *Aldh1a1*, *Wt1*, *Col4a1*, and *Ccl19* in naive and activated states for male and female mouse OM. **(C)** qRT-PCR validation of *Aldh1a1*, *Wt1*, *Col4a1*, and *Ccl19* expression levels in male and female OM. **(D)** UMAP plots illustrating expression of chemokine *Cxcl13* and *Ctsb* in mouse OM. **(E)** qRT-PCR quantification of *Cxcl13* expression levels between male and female OM.

To comprehensively characterize non-immune cells from the male and female mouse omentum, we next performed detailed comparative analysis of non-immune scRNA-seq data. Omental stromal cells were classified into two distinct subtypes: mesothelial cells (*Enpp2* ^+^) and fibroblasts (*Lum* ^+^). Mesothelial cells exhibited high expression of retinoic acid-related genes, including Wilms tumor 1 transcription factor (*Wt1*) and aldehyde dehydrogenase 1 family member A1 (*Aldh1a1*), while fibroblasts predominantly expressed extracellular matrix-associated genes, such as collagen type iv alpha 1 chain (*Col4a1*) and laminin subunit alpha 4 (*Lama4*), along with the T-cell chemokine c-c motif chemokine ligand 19 (*Ccl19*) (**Figure 5A**, **Supplementary Figure 6B**). Notably, male mesothelial cells displayed significantly higher expression of the retinol dehydrogenase gene *Aldh1a1* compared to their female counterparts (**Figure 5B**). Retinoic acid, the metabolic product of Aldh1a1 enzymatic activity, is known to play a crucial role in maintaining transforming growth factor beta 1(*Tgf-β*)^+^ *Gata6* ^+^ peritoneal macrophages.

To validate these findings experimentally, non-immune cells (CD45-negative) were isolated from the omentum via fluorescence-activated cell sorting (FACS), followed by qRT-PCR analysis. Consistent with scRNA-seq results, *Aldh1a1* mRNA expression was significantly elevated in male omental non-immune cells relative to females, regardless of inflammatory status (**Figure 5C**). Interestingly, among other examined genes, no substantial sex differences were observed.

Additionally, our analysis showed that omental mesothelial cells constitutively expressed the chemokine *Cxcl13*. However, *Ctsb*, a key enzyme responsible for Cxcl13 release, was exclusively expressed in omental macrophages, indicating a critical collaborative role between omental macrophages and stromal cells in regulating B-cell chemotaxis within the omentum (**Figure 5D**).

### 2.6 Omental macrophage characteristics facilitate ovarian cancer metastasis

The omentum is recognized as the most frequent metastatic site for ovarian cancer, which typically originates from the ovary or fallopian tube and subsequently spreads throughout the peritoneal cavity, ultimately colonizing the omentum (26). The majority of women diagnosed with high-grade serous ovarian carcinoma exhibit metastatic lesions within the omentum, underscoring its critical clinical relevance (27). Prior studies identified a specific subset of omental resident macrophages, characterized by co-expression of *Cd163* and *Tim4*, which establishes a favorable niche for ovarian cancer metastasis (10). However, the precise spatial distribution and quantitative enrichment of these macrophages remain incompletely understood.

**Figure 6:**
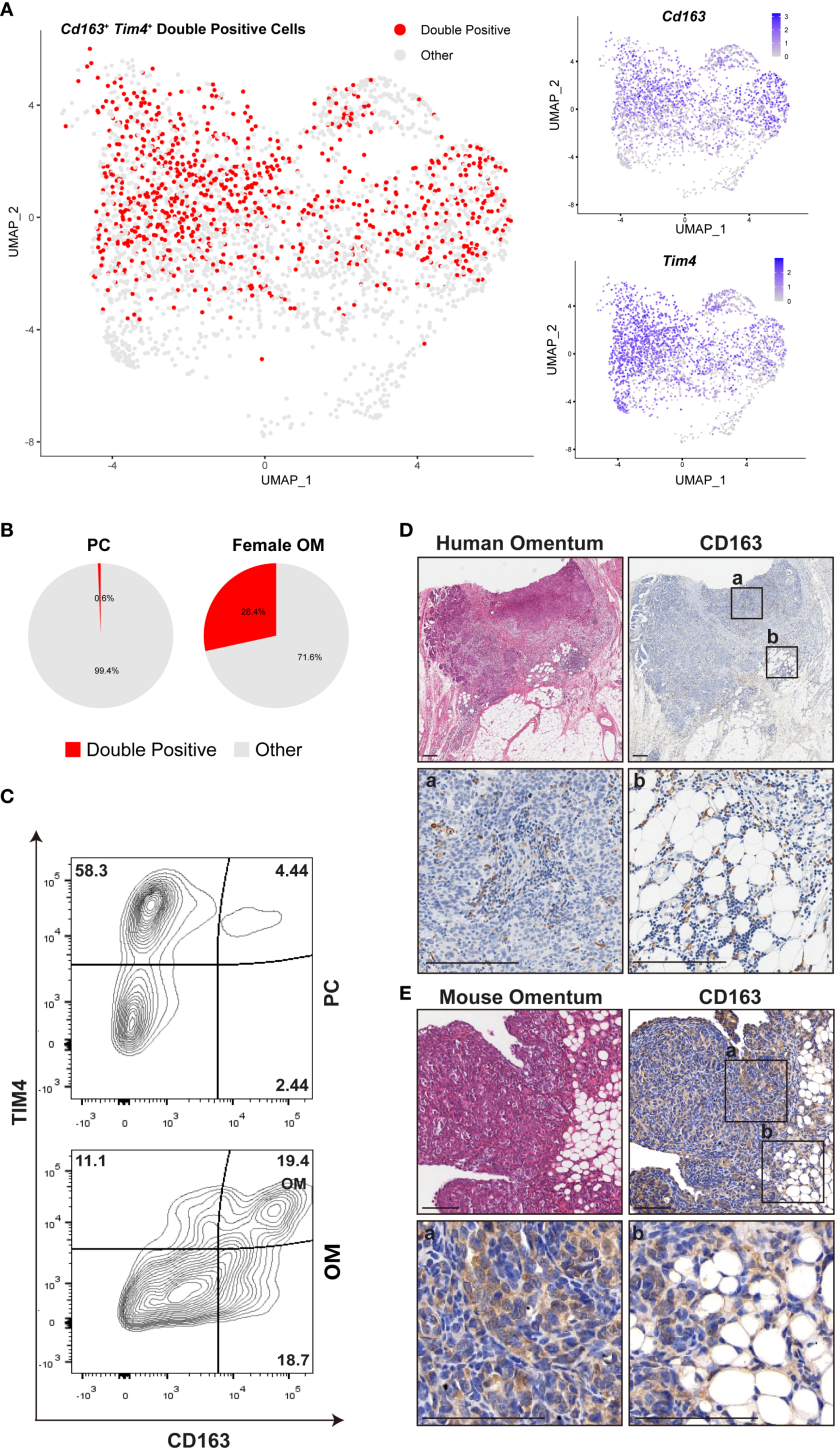
Female omental microenvironment determines susceptibility to ovarian cancer metastasis. **(A)** UMAP plots displaying expression of *Cd163*, *Tim4*, and their co-expression (double-positive cells) within female mouse omentum OM. **(B)** Comparative quantification of *Cd163* ^+^ *Tim4* ^+^ double-positive macrophages between peritoneal cavity (PC) and female OM. **(C)** Flow cytometry analysis of CD163^+^ TIM4^+^ macrophages isolated from PC and OM, gated on live CD45^+^ CD11b^+^ cells. **(D, E)** Representative images of H&E and IHC staining for CD163 in human and mouse omental tissues. Labels “a” and “b” denote the locations of milky spots and omental margins, respectively. Scale bars: 200 μm (human tissues in **D**) and 100 μm (mouse tissues in **E**).

To address this knowledge gap, we utilized our scRNA-seq dataset to specifically analyze the *Cd163^+^Tim4^+^* macrophage population, aiming to delineate their spatial distribution and expression patterns within the omentum (**Figure 6A**). Our analysis revealed a significant enrichment of these double-positive macrophages predominantly within the omentum compared to the PC (**Figure 6B**, **Supplementary Figures 7A-C**).

We next conducted comparative quantitative analyses between CD163^+^TIM4^+^ macrophage populations in female omentum and peritoneal cavity samples. Flow cytometry analysis further confirmed that the proportion of CD163^+^ TIM4^+^ macrophages was significantly higher within the female omentum compared to the peritoneal cavity (**Figure 6C**), clearly indicating tissue-specific enrichment.

To further validate the spatial localization and biological relevance of these macrophages in the context of ovarian cancer metastasis, we performed H&E staining and IHC for CD163 on mouse and human omental tissues bearing ovarian cancer metastases. Consistent with our transcriptomic data, CD163^+^ macrophages were abundantly detected within metastatic sites in the omentum, strongly suggesting their critical role in promoting ovarian cancer dissemination and colonization (**Figures 6D, E**). Insets labeled “a” and “b” within **Figures 6D, E** represent distinct anatomical regions of the omentum: inset “a” specifically highlights immune cell-rich milky spots, whereas inset “b” indicates the peripheral edge region of the omentum.

Collectively, these findings clearly demonstrate that CD163^+^ TIM4^+^ macrophages in the omentum represent a distinct tissue-specific cellular subset crucial for ovarian cancer metastasis, highlighting their potential as therapeutic targets or prognostic biomarkers for ovarian cancer.

## 3 Discussion

The greater omentum, widely recognized as the “abdominal policeman,” is a visceral adipose tissue playing essential immunological roles in regulating peritoneal homeostasis. Structurally, it comprises specialized fat-associated lymphoid clusters (FALCs) and mesothelial cells critical for preventing peritoneal inflammation (28). Recent findings have highlighted the sexually dimorphic characteristics of peritoneal macrophages, primarily shaped by differences in the peritoneal microenvironment (29). Specifically, the male peritoneal cavity remains relatively isolated, while the female peritoneal cavity communicates externally via the fallopian tubes and uterine cavity, potentially making females more susceptible to peritoneal infections. However, until now, it remained unclear whether similar sexual dimorphisms existed within the omentum itself.

ScRNA-seq enables high-resolution characterization of cellular heterogeneity beyond traditional bulk RNA sequencing methods. Leveraging this approach, we systematically analyzed the cellular composition and functional features of male and female mouse omentum under both physiological and inflammatory conditions (30). Initially, we defined three functionally distinct macrophage subsets within the omentum: inflammatory macrophages, resident macrophages, and M2-like macrophages. Notably, these subsets exhibited dynamic proportional shifts upon inflammation, marked by increased inflammatory macrophages and decreased resident and M2-like macrophages, highlighting the adaptability of macrophage subsets during inflammatory responses. We further elaborated on the specialized functional transitions among these macrophage subsets, clearly delineating how their dynamic changes facilitate immune regulation, tissue repair, and inflammation resolution. These insights were carefully integrated with previous literature examining macrophage functional plasticity in other tissues, such as adipose tissue and the peritoneum, thereby underscoring the complementary and expansive nature of our findings (28–30).

We have additionally identified significant transcriptional differences between macrophages in the omentum and those within the peritoneal cavity. For instance, peritoneal macrophages specifically expressed *Gata6* and *Thbs1*, which were absent in omental macrophages. Conversely, omental macrophages uniquely expressed adipose tissue-associated markers such as *Mrc1* and *Retnla*, suggesting their specialized roles in maintaining adipose tissue homeostasis. Additionally, key retinoic acid metabolism genes (*Wt1* and *Aldh1a1*), critical for sustaining *Gata6* ^+^ peritoneal macrophages (31), were notably absent in peritoneal cells but highly expressed in *Enpp2* ^+^ stromal cells within the omentum. This observation strongly suggests cross-tissue regulatory interactions, where omental stromal cells potentially support peritoneal macrophage functions via retinoic acid production. This clearly demonstrates significant tissue-specific transcriptional differences between omental and peritoneal macrophages, expanding upon previous knowledge regarding macrophage functions in distinct tissue environments and emphasizing the critical influence of tissue microenvironment on macrophage functional identities. Additionally, our findings explicitly identify a previously unrecognized role for omental stromal cells in sustaining peritoneal macrophage function.

Moreover, our analysis revealed significant sexually dimorphic gene expression within omental stromal cells. Male mesothelial cells displayed elevated *Aldh1a1* expression compared to females, yet intriguingly, this higher retinoic acid production did not markedly affect macrophage functions within the omentum itself, but instead significantly influenced peritoneal macrophage polarization. Potential explanations for this phenomenon include: (1) differential expression of retinoic acid receptors between omental and peritoneal macrophages leading to variable sensitivity; (2) differences in local tissue microenvironments influencing retinoic acid diffusion and activity; and (3) distinct steady-state or activation statuses of macrophages across tissues determining their retinoic acid responsiveness. Future studies utilizing tissue-specific genetic knockout models or retinoic acid receptor conditional deletion could provide mechanistic clarity regarding these hypotheses. Furthermore, we identified two functionally divergent stromal subsets (*Lum* ^+^ and *Enpp2* ^+^), involved in extracellular matrix remodeling and immune modulation or tumor promotion, respectively, emphasizing the complexity and versatility of stromal cell functions under both physiological and pathological conditions (32, 33).

Critically, in the context of ovarian cancer metastasis, omental macrophages exhibited specific transcriptional signatures associated with tumor migration and invasion, highlighting their functional plasticity in pathological states from protective immune roles toward facilitating tumor progression. Clinically, these findings underscore the potential of targeting specific macrophage populations or their stromal regulators as novel therapeutic strategies or biomarkers for ovarian cancer metastasis, which merits further translational exploration.

Despite these advancements, certain limitations persist. ScRNA Seq methods inherently suffer from transcriptional dropout, potentially leading to sporadic or undetected expression of certain key marker genes. For instance, we observed limited expression of *Ptprc* (CD45) in some immune cell subsets (**Supplementary Figure 2A**). While *Ptprc* is a well-known pan-immune marker, its expression can vary across different immune cell types or activation states, with certain subsets (such as plasma cells or activated T cells) known to downregulate *Ptprc* expression. Additionally, technical factors inherent to scRNA-seq methodologies might also contribute to reduced detection levels. To address these concerns, we used combinatorial analyses of multiple well-established lineage-specific markers (e.g., *Ms4a1*, *Cd3g*, *Cd79b)* rather than relying solely on *Ptprc* expression, thereby ensuring robust and accurate cell subset classification. However, caution remains essential when interpreting cell-type classifications based on single marker genes or low-expression transcripts. Furthermore, the precise origins, recruitment mechanisms, and clinical significance of the identified cell populations warrant additional detailed exploration in future studies.

In summary, our study provides a comprehensive characterization of omental cellular heterogeneity across sex, inflammatory conditions, and pathological contexts, highlighting previously unappreciated cross-tissue interactions between omental stromal cells and peritoneal macrophages. These insights deepen our understanding of peritoneal immune regulation and reveal potential therapeutic targets and biomarkers for inflammatory and metastatic peritoneal diseases. Future research should focus on defining the cellular origins, recruitment mechanisms, and clinical implications of these findings, which are active areas of investigation in our ongoing studies.

## 4 Materials and methods

### 4.1 Animals

Mice were housed and bred according to protocols approved by the East China Normal University Animal Care Commission. Specifically, animals were housed in a controlled environment with 12-hour light/dark cycles and provided with a standard diet and water ad libitum. All animals received humane care according to the criteria outlined in the “Guide for the Care and Use of Laboratory Animals” prepared by the National Academy of Sciences and published by the National Institutes of Health.

Adult male and female C57BL/6 mice (8–10 weeks old) were used for isolation of single cells from mouse omentum for single cell RNA-seq analysis.

For the activation of omentum, C57BL/6 mice were injected intraperitoneally with 1 ml polydextran bead slurry (Sephadex^®^ G-25, G25150, Sigma) (1:1 in normal saline). Seven days after injection, mice were euthanized by gradual-fill CO_2_ inhalation, followed by cervical dislocation to ensure death, in accordance with institutional ethical guidelines. The omenta were then collected immediately for downstream analysis.

### 4.2 Preparation of single cells from the omentum

For analysis under physiological conditions, omenta from six male or six female mice were pooled to generate single-cell suspensions. For analyses under inflammatory conditions, omenta from two to three male or female mice were similarly pooled. Briefly, omenta were finely minced and digested continuously in collagenase I (Sigma, C0130) at 37 °C. Single-cell suspensions were sequentially collected at three distinct digestion intervals (after 10, 20, and 30 minutes), maximizing the recovery of diverse cell types that might be released at different digestion durations. Following digestion, the collected cells were washed with serum-free medium and filtered through a 40 µm cell strainer to obtain a purified single-cell suspension suitable for subsequent analyses.

### 4.3 Single cell RNA-sequencing

Approximately 10,000 to 15,000 single cells from both naive and activated omentum were used to generate scRNA-seq libraries, which were subsequently sequenced and analyzed. Single cells from each sample were encapsulated in droplets using 10x Genomics Gemcode Technology and processed according to the manufacturer’s protocols (#CG00052). The cDNA and libraries were assessed for quality using an Agilent 4200 Tapestation and quantified by KAPA qPCR prior to sequencing on a single lane of a HiSeq4000 (Illumina).

### 4.4 Single cell RNA-sequencing data analysis

For the scRNA-Seq datasets of mouse omentum, detailed information on the number of cells and genes was obtained for further analysis, as presented in **Supplementary Table 1**. Highly variable genes were identified using the MeanVarPlot function in Seurat with default settings, aiming to select the top approximately 2,000 variable genes. Single-cell data clustering was performed using a graph-based approach in Seurat, with the resolution parameter in the FindClusters function set to 0.8. Clusters were visualized using a unified manifold approximation and projection (UMAP, version 0.2.6.0) plot. Data analyses were conducted using the R package Seurat (version 2.3.4).

During quality control, cells with UMI counts below 500 and potential doublets were removed. Additionally, cells with a mitochondrial gene percentage exceeding 5% or a ribosomal gene percentage exceeding 30% were filtered out. For the integration analysis of single-cell data, samples from mouse omentum, peritoneal cavity (GSE124562), and omentum stroma (GSE136636) were normalized using the SCTransform method and integrated using reciprocal principal component analysis (PCA) (https://satijalab.org/seurat/v3.1/integration.html) (34). The integrated dataset was further subjected to PCA and cluster analysis using UMAP at a resolution of 0.2. Differential expression genes (DEGs) were detected using volcano plots generated with the R package ggplot2 (version 3.3.2).

### 4.5 H&E and immunohistochemistry staining

H&E and IHC staining were performed as described previously (35).

### 4.6 Immunofluorescence staining

All human tissues were obtained with informed consent, and the study was approved by the Research Ethics Committee of Fengxian District Center Hospital. For tissue immunofluorescence analysis, paraffin sections (5μm) of human tumors were deparaffinized, rehydrated with graded ethanol, and subjected to antigen retrieval. The samples were blocked with 10% donkey serum (Sigma-Aldrich, D9663, USA) for 1 hour at room temperature, followed by incubation with primary antibodies overnight at 4 °C. After washing three times with PBS, the slides were incubated with secondary antibodies for 30 minutes at room temperature. Nuclei were stained with 4′,6-diamidino-2-phenylindole (DAPI). Digital images were captured using fluorescence or confocal microscopes equipped with a digital camera (Nikon, Japan). Detailed information on the primary and secondary antibodies used in this assay is provided in **Supplementary Table 2**.

### 4.7 Isolation and purification of macrophages

For the mouse peritoneal macrophages, mice were killed and the cells were obtained by lavage of the peritoneal cavity with ice-cold PBS via a plastic catheter as previously described (36). For the mouse omentum macrophages, the cells were obtained after enzyme digestion. Then the cells were labeled with rat Alexa Fluor 488-conjugated anti-mouse F4/80 antibodies, and F4/80^+^ macrophages were sorted using Aria-II (BD) FACS. The sorted macrophages either from peritoneum or omentum were used for qPCR analysis.

### 4.8 Flow cytometry and FACS sorting

Single cells isolated from mouse omentum or peritoneum were incubated with fluorochrome-conjugated antibodies at the recommended dilutions or with isotype control antibodies for 20 minutes at 4 °C. The stained cells were then analyzed by flow cytometry using a BD LSR-Fortessa cell analyzer (BD). FlowJo v.10.4.2 software (BD) was used for further analysis. Detailed information on the antibodies used in this assay is provided in **Supplementary Table 2**.

### 4.9 Real time PCR

Total RNA extraction and reverse transcription were performed using Trizol reagent (Takara) and the PrimeScript RT-PCR kit (Takara) according to standard protocols. Real-time PCR was employed for gene expression analysis, and SYBR Premix Ex Taq (Roche) was used on a 7500 Real-time PCR system (Applied Biosystems) with the recommended thermal cycling conditions. Relative mRNA expression levels were calculated using the 2^(-ΔΔCt)^ method and normalized to β-actin mRNA levels. The primer sequences used in this study are provided in **Supplementary Table 3**.

### 4.10 Statistical analysis

Data were presented as the means ± standard error of mean (SEM). Statistical analyses were done using GraphPad Prism 5 software (GraphPad Software Inc., San Diego, CA). Student’s T-test was used for comparison between groups. Values of P<0.05 were considered statistically significant.

## Data Availability

All single-cell RNA sequencing data are available in the Gene Expression Omnibus (GEO) under accession number GSE212419. The remaining data supporting the findings of this study are available within the article and its Supplementary Information files or from the corresponding authors upon reasonable request.
