# Supplementary material for: Identification of sex- and inflammation-associated heterogeneity in the mouse omentum
Source: Front Immunol. 2025 Oct 10;16:1670112. doi: 10.3389/fimmu.2025.1670112 (PMC12589825; doi:10.3389/fimmu.2025.1670112)
Supplement: Supplementary file 1 [file Table1.docx]

*Supplementary Material*

**
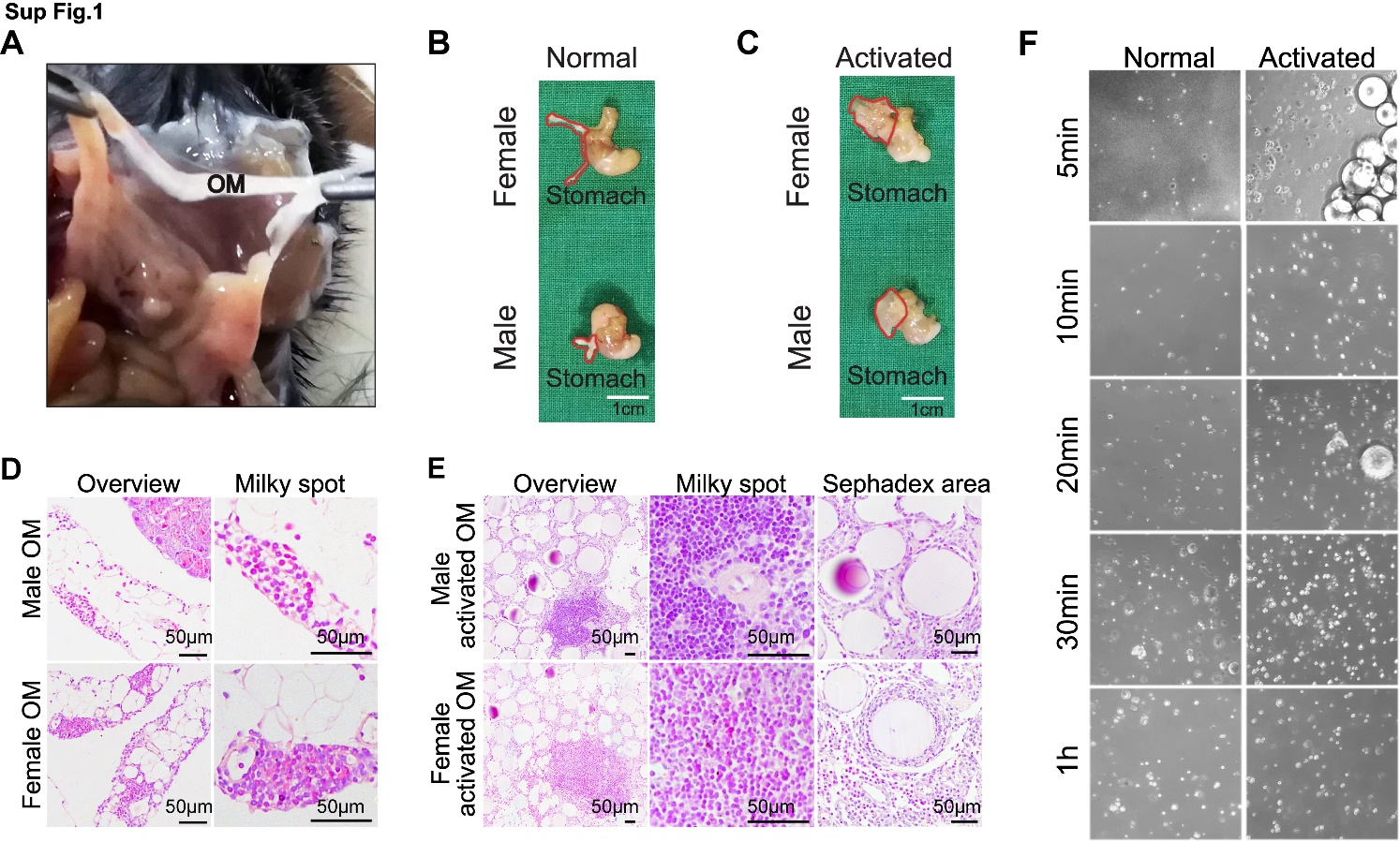
**

**Supplementary Fig.1 The macroscopic & microscopic images of mouse omentum.** (A). The anatomy location of the omentum in the mouse peritoneal abdominal cavity. (B-C) The gross morphology of the mouse omentum in both male and female mouse with or without activation. Scale bar = 1cm. (D-E) HE staining of mouse omentum in both male and female mouse with or without activation. Scale bar =50μm. (F) The microscopic images of single cells from mouse omentum at different time points after collagenase I digestion.

**
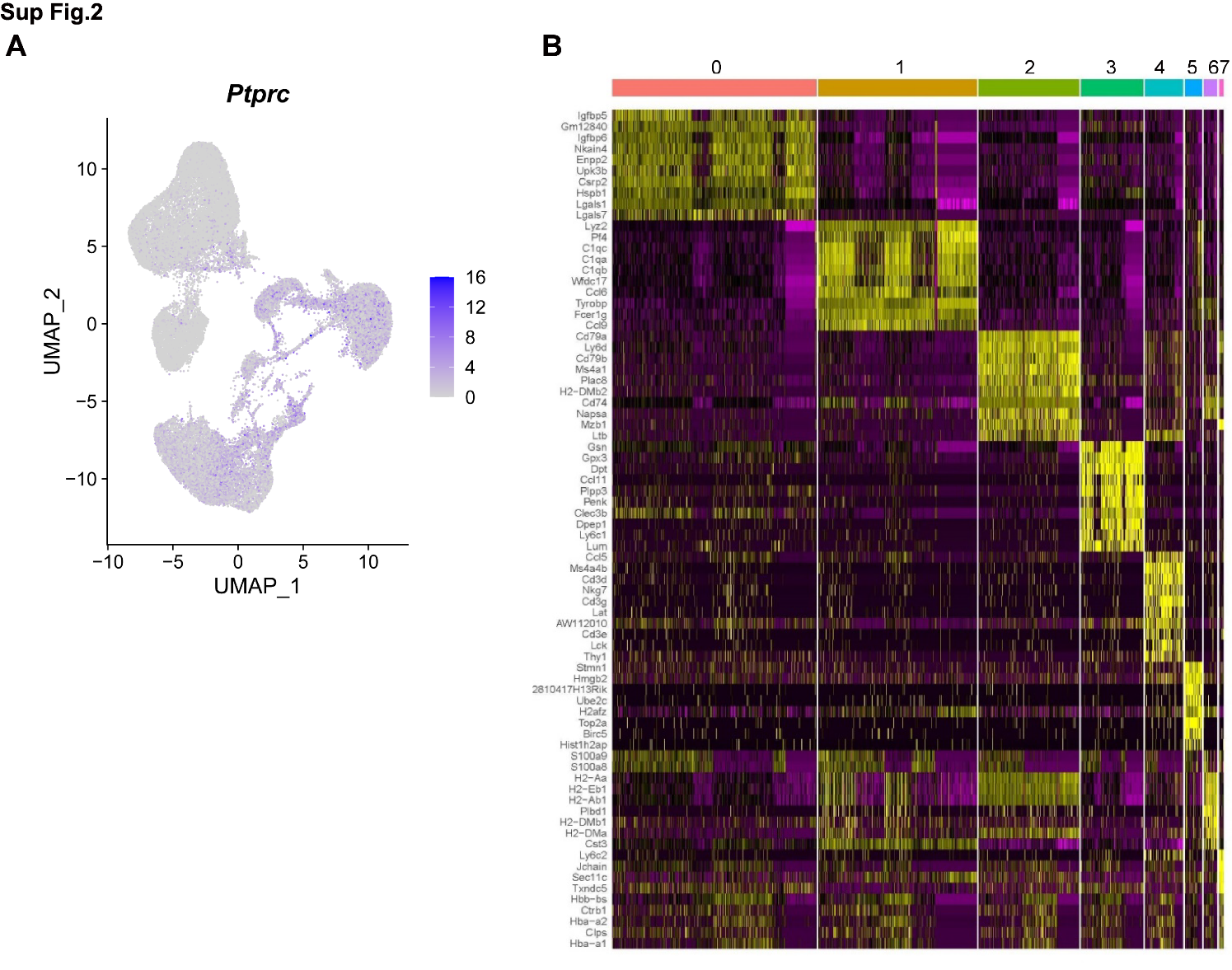
**

**Supplementary Fig.2 Single cell RNA-sequencing analysis of mouse female and male omentum.** (A) *Ptprc* gene expression distinguishing the immune cells and non-immune cells projected onto UMAP plots. Color scaled for each gene with highest log-normalized expression level noted. (B) Heatmap showing the expression of differentially expressed genes (DEGs), which represent Top 10 genes in one cluster compared to other clusters.


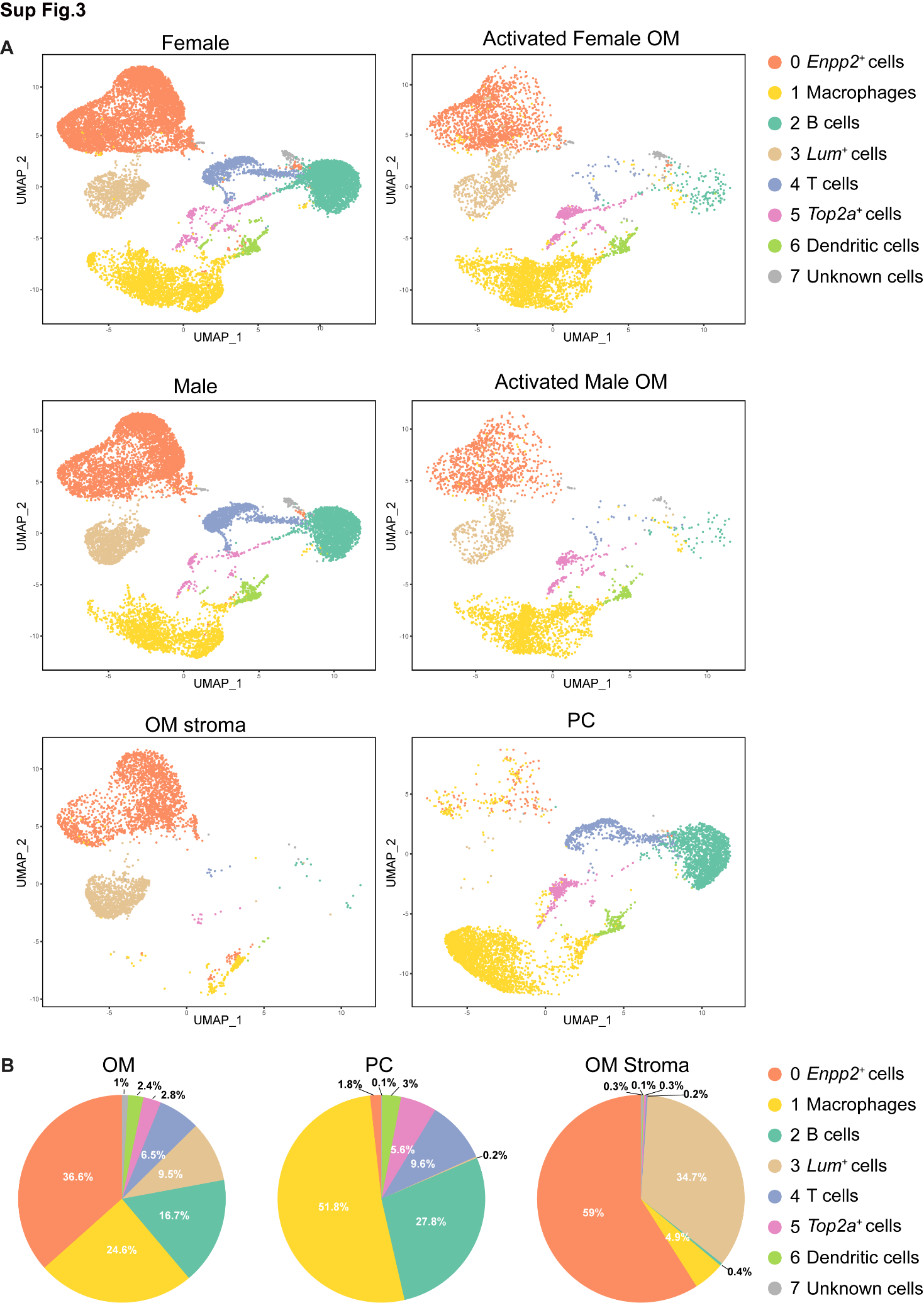


**Supplementary Fig.3 Single cell RNA-sequencing analysis for different mouse tissues.**  (A) Unsupervised clustering of the single cells from mouse female omentum (OM), activated female OM, male OM, activated male OM, OM stroma and peritoneal cavity (PC) visualized with UMAP. (B) Percentage of different cell clusters in mouse OM, PC and OM stroma.

**
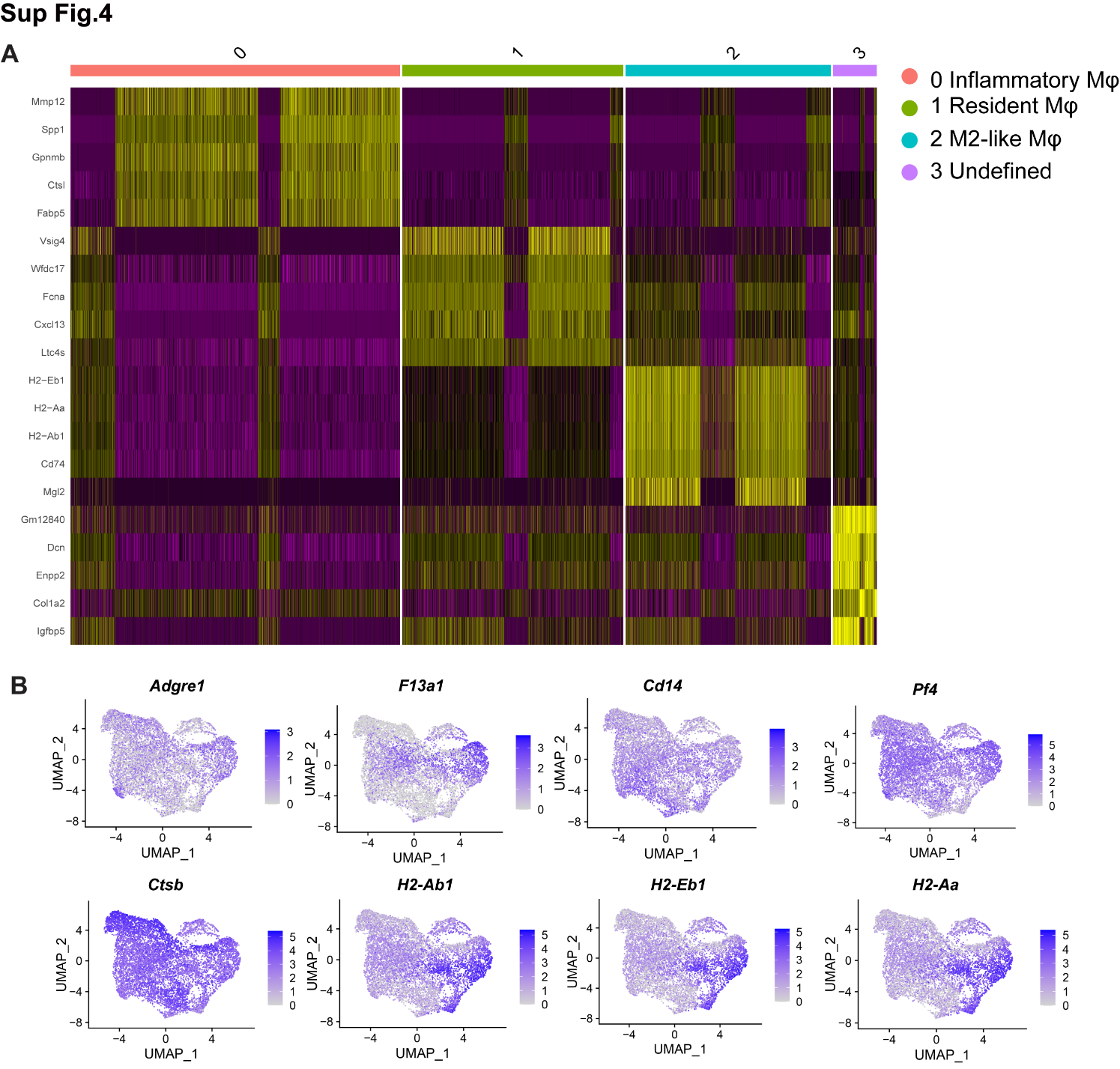
**

**Supplementary Fig.4 Macrophages subtypes in mouse omentum in naïve and inflammatory situations.** (A) Heatmap showing the expression of differentially expressed genes (DEGs) in different macrophage clusters, which represent Top 10 genes in one cluster compared to other clusters. (B)Macrophages-related gene expression distinguishing the 3 clusters projected onto UMAP plots. Color scaled for each gene with highest log-normalized expression level noted.


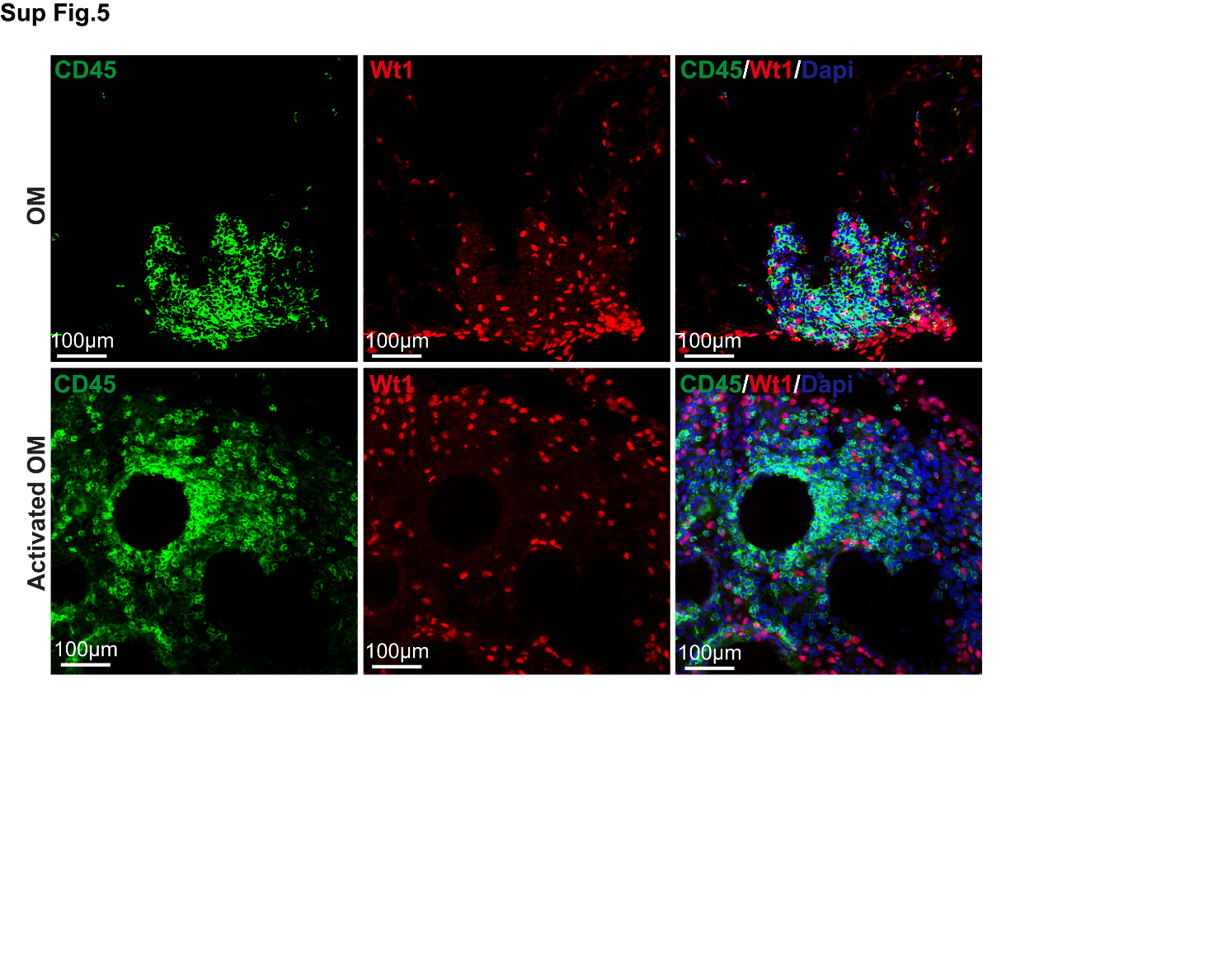


**Supplementary Fig.5 IF staining of mouse omentum in different status.**  IF staining of mouse omentum in naïve and inflammatory situations showing the presence of immune cells (*Cd45*) and non-immune cells (*Wt1*). Dapi indicated nuclei. Scale bar =100 μm.


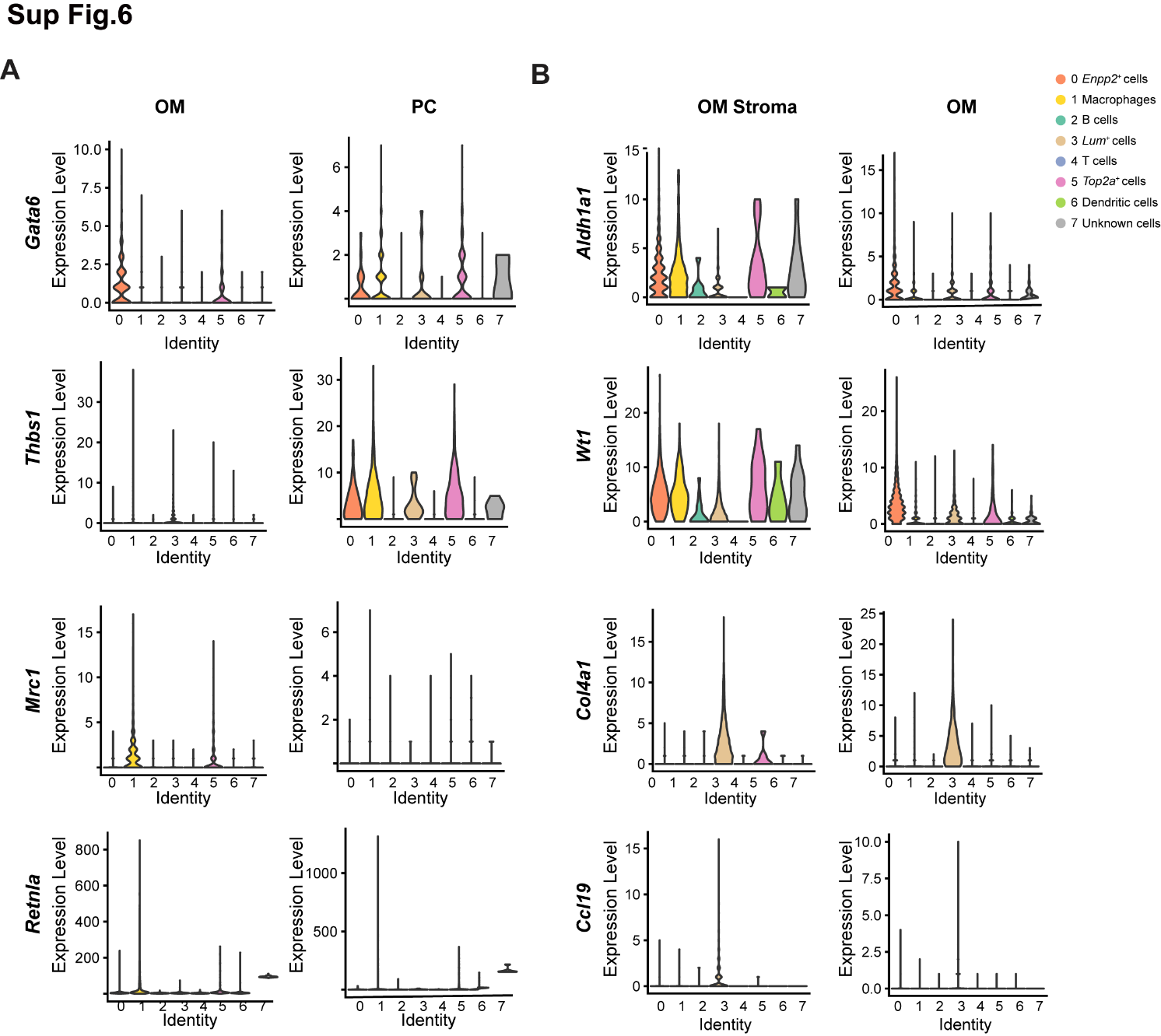


**Supplementary Fig.6 Violin plots illustrating expression patterns of selected marker genes.** (A) Violin plots displaying the expression distributions of macrophage-associated marker genes (*Gata6, Thbs1, Mrc1, Retnla*) in macrophages isolated from omentum (OM) and peritoneal cavity (PC). (B) Violin plots showing gene expression patterns of stromal cell-associated markers (*Aldh1a1, Wt1, Col4a1, Ccl19*) within omental stroma (OM Stroma) and OM. Cell clusters are indicated by different colors according to cell identity annotation.


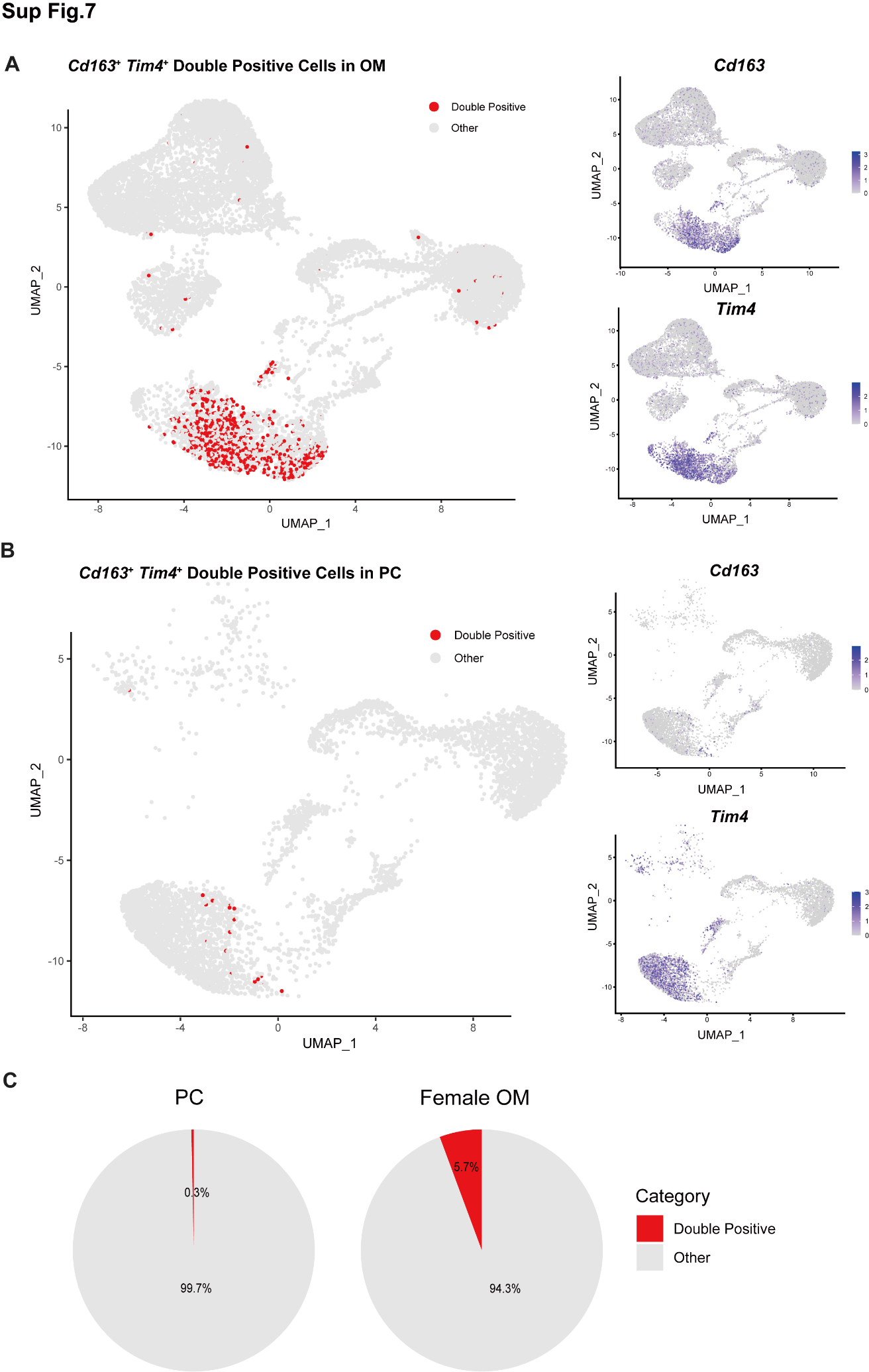


**Supplementary Fig.7 Analysis of *Cd163*^+^ *Tim4*^+^ cells in female OM and PC.** (A-B) *Cd163* and *Tim4* gene expression in OM (A) and PC (B) projected onto UMAP plots. (C) The percentage of *Cd163*^+^ *Tim4*^+^ cells among total cells in both PC and female OM.

**Supplementary Table.1** The information for RNA-Seq analysis data of mouse omentum

| **Sex** | **Status** | **No. of genes** | **No. of cells** | | |
| --- | --- | --- | --- | --- | --- |
|  |  |  | **Original** | **Remove>5% mit and>30% rib** | |
| Female | Naïve | 18307  17927  17841  16436 | 15875 | | 9785 |
| Male | Naïve |  | 5699 | | 4496 |
| Female | Active |  | 13357 | | 8827 |
| Male | Active |  | 3974 | | 3176 |

**Supplementary Table.2** The antibodies used for flow cytometry analysis

| **Antigen** | **Clone** | **Dye** | **Catalog number** | **Supplier** | **Purpose** |
| --- | --- | --- | --- | --- | --- |
| CD45.2 | 30-F11 | AF700 | 1800-27 | BD Pharmingen | FC |
| CD11b | M1/70 | Brilliant Violet 605™ | 101257 | Biolegend | FC |
| F4/80 | BM8 | Alexa Fluor® 488 | MF48020 | Invitrogen™ | FC |
| CD163 | TNKUPJ | PE | 12-1631-82 | eBioscience™ | FC |
| Tim4 | RMT4-54 | Alexa Fluor® 647 | 130008 | Biolegend | FC |
| 7AAD |  |  | 00-6993-50 | Invitrogen™ | FC |
| CD45 | 30-F11 |  | NB100-77417 | Novus biologicals | IF |
| CD31 | 900 |  | 102401 | Biolegend | IF |
| Arg1 | polyclonal |  | ab60176 | Abcam | IHC/IF |
| CD163 | EPR19518 |  | ab182422 | Abcam | IHC |
| S100a8/a9 | MAC387 |  | ab22506 | Abcam | IHC |
| Wt1 | CAN-R9(IHC)-56-2 |  | ab89901 | Abcam | IHC/IF |
| F4/80 | BM8 |  | ab16911 | Abcam | IHC/IF |

FC: flow cytometry; IHC: immunohischemistry; IF: immunofloresence.

**Supplementary Table.3 The sequences of primers used in this study**

| Gene | Forward primer (5’-3’) | Reverse primer (5’-3’) |
| --- | --- | --- |
| *Wt1* | GAGAGCCAGCCTACCATCC | GGGTCCTCGTGTTTGAAGGAA |
| *Ccl19* | GGGGTGCTAATGATGCGGAA | CCTTAGTGTGGTGAACACAACA |
| *Col4a1* | CTGGCACAAAAGGGACGAG | ACGTGGCCGAGAATTTCACC |
| *Gata6* | GCCGGAGGAAATGTACCAGAC  AG | CCCCTTGAAGGTAGGGCAG  TC |
| *Thbs1* | GGGGAGATAACGGTGTGTTTG  TAC | CGGGGATCAGGTTGGCATT  AC |
| *Mrc1* | CTCTGTTCAGCTATTGGACGC  GCTC | CGGAATTTCTGGGATTCAGCTTC |
| *Retnla* | CCAATCCAGCTAACTATCCCTCC  ACCCAGTAGCAGTCATCCCA | ACCCAGTAGCAGTCATCCCA |
| *Aldh1a1* | ATACTTGTCGGATTTAGGAGGCT | GGGCCTATCTTCCAAATGAACA |
| *Cxcl13* | GGCCACGGTATTCTGGAAGC | GGGCGTAACTTGAATCCGATCTA |
| *Cstb* | TCCTTGATCCTTCTTTCTTGCC | ACAGTGCCACACAGCTTCTTC |
| *Tnfrsf13b* | ATGGCATTCTGCCCCAAAGAT | ATGGTCGTAGTACCTGCCTTG |
| *Itga6* | TGCAGAGGGCGAACAGAAC | GCACACGTCACCACTTTGC |
| *Cd163* | ATGGGTGGACACAGAATGGTT | CAGGAGCGTTAGTGACAGCAG |
| *Actb* | GGCTGTATTCCCCTCCATCG | CCAGTTGGTAACAATGCCATGT |
